# Dose-Dependent Alterations to In Vitro Human Microbiota Composition and Butyrate Inhibition by a Supercritical Carbon Dioxide Hops Extract

**DOI:** 10.3390/biom9090390

**Published:** 2019-08-21

**Authors:** Paul A. Blatchford, Shanthi G. Parkar, Wendy Hopkins, John R. Ingram, Kevin H. Sutton

**Affiliations:** 1 The New Zealand Institute for Plant and Food Research Limited (PFR), Private Bag 11600, Palmerston North 4442, New Zealand; 2 PFR, Private Bag 3123, Hamilton 3240, New Zealand; 3 PFR, Private Bag 92169, Auckland 1142, New Zealand; 4 PFR, Private Bag 4704, Christchurch 8140, New Zealand

**Keywords:** Pacific Gem hops extract, alpha acids, beta acids, in vitro human fecal fermentation, butyric acid, *Bifidobacterium*, *Akkermansia*, Enterobacteriaceae, Lachnospiraceae

## Abstract

Hop cones (*Humulus lupulus* L.) have been used throughout history as an additive in beer brewing and as herbal supplements with medicinal and culinary properties. The objective of this study was to ascertain the effect of a range of concentrations of a supercritical CO_2_ extract of hops on the composition and metabolism of human gut bacterial communities using *in vitro* batch culture systems. Fermentations were conducted over 24 h using a mixed human fecal inoculum. Microbial metabolism was assessed by measuring organic acid production and microbial community alterations were determined by 16S rRNA gene sequencing. Butyrate, an important short chain fatty acid in maintaining colonic well-being, decreased at elevated concentrations of hops, which may partly be accounted for by the concomitant reduction of *Eubacterium* and *Coprococcus*, known butyrate-producing genera, and also the inhibition of *Bifidobacterium*, a beneficial organism that has a butyrogenic effect through metabolic cross-feeding with intestinal commensals. The hops compounds also caused dose-dependent increases in the potentially pathogenic Enterobacteriaceae and potentially beneficial *Akkermansia*. Thus, hops compounds had a significant impact on the structure of the bacterial consortium, which warrants further study including human clinical trials.

## 1. Introduction

The gastrointestinal microbiota is a highly adaptive collection of microorganisms that have evolved over millennia to a state of equilibrium within its human host. Harboring several orders of magnitude more genetic material than the human genome has enabled the microbiota to exploit endogenous, host and residual dietary substrates to maintain a prominent niche in the colonic environment [1,2]. The main end products of colonic fiber fermentation are organic acid metabolites such as acetate, propionate and butyrate. These acids serve as metabolic cross feeders to boost the growth of beneficial bacteria and even prevent colonization by opportunistic pathogens. Through direct (binding, infiltrating host epithelia) and indirect (microbial metabolites, lipopolysaccharides) mechanisms, the composition and metabolism of the gut microbiota can profoundly influence host health and immune status. Therefore, maintaining a diverse, pathogen-free, commensal-enriched microbiota is an important target for avoiding dysbiosis and associated gastrointestinal disturbances.

Typical approaches used for improving the microbiome composition are through probiotics, prebiotics and synbiotics or through increasing dietary fiber intake [3,4]. A less common consideration regarding microbiome health is the inhibitory or antimicrobial effect of constituents of our diet. Many plants contain antimicrobial compounds that help them resist infection by microorganisms in nature and these can persist after cooking and digestion to reach the large bowel where their antimicrobial properties can alter microbiota composition [5,6]. One such commonly consumed plant is hops (*Humulus lupulus* L.), which are primarily used in the brewing industry as bittering and flavoring additives, with the further benefit of inhibition of unwanted spoilage microorganisms. Hops are also used in the medicinal, cosmetic and culinary fields and are considered as an alternative to antibiotics in animal feed [7]. The inhibitory action of hops acids, purportedly through membrane perturbation and dissipation of intracellular ions, is typically observed against Gram-positive bacteria with limited evidence of efficacy against Gram-negative microorganisms [8,9]. The primary antimicrobial components in hops extracts are the bitter α- and β-acids, which are prenylated phloroglucinol derivatives, concentrated in the strobiles (seed-bearing, cone-like structures). The α-acids include humulone and its congeners cohumulone, adhumulone, prehumulone and posthumulone. The β-acids include lupulone and its congeners colupulone, adlupulone, prelupulone and postlupulone. These terpenoid compounds are hydrophobic, oily substances that evade human digestive enzymes and acids, thereby reaching the large bowel intact and so are able to interact with the resident colonic microbiota [10]. Supercritical carbon dioxide extraction is a well-established method for extraction of these hops α- and β-acids with the extract shown to possess antimicrobial activity [11,12]. Considering that hops compounds are digestion-resistant and antibacterial as well as being used extensively in the food, beverage and nutraceutical industries, it is reasonable to interrogate the effect of hops compounds on the human colonic microbiota. Batch culture gut models provide a scientifically validated simulation of the colonic environment that gut microbiota inhabit, and which is amenable to experimental investigation of effects of plant foods on gut microbiota [13]. Several studies have been conducted in chickens to explore the feasibility of hops use as an alternative to antibiotics in feed and these have generally observed minimal effects on microbiota composition [14,15]. Other research describes the effect of compounds present in hop extracts, such as xanthohumol, on single strains of bacteria and concluded that these have a pronounced inhibitory effect, mainly on Gram-positive bacteria (*Bacillus, Streptococcus, Staphylococcus*, etc.) [8,16]. Gut microbiota mediated biotransformation products of xanthohumol and isoxanthohumol have been identified as having estrogenic bioactivity using in vitro fermentation systems [10,17,18]. This is the first study to evaluate the effect of a supercritical CO_2_ hops extract on human fecal microbiota using batch culture gut models. As hops is becoming a more common ingredient in our diets, this study bears relevance for human gut health and wellbeing, and will help to inform on the development of functional foods and nutraceuticals containing hops compounds.

## 2. Materials and Methods

### 2.1. Hops Composition

The hops extract (*Humulus lupulus*—cultivar “Pacific Gem”), which was prepared using supercritical CO_2_ extraction, was kindly supplied by NZ Hops Ltd., Nelson, New Zealand. The detailed composition of the hops extract was determined by high performance liquid chromatography (HPLC) with reference to the American Brewing Association ICE-3 standard. In order to reduce the viscosity of the hops extract, and to aid its dispersibility in the in vitro digestion system, the hops extract was mixed with commercially obtained canola oil in (at least) a 2:1 (hops: oil) ratio. The amount of canola oil was kept constant in the fermentation vessels and an oil-only control was included (Table 1).

### 2.2. In Vitro Fermentations

Fermentation was conducted in triplicate using the pH-controlled anaerobic batch fermenters as described in a previous study [19], with minor modifications. The base culture media used was a minimal media, with 0.2% galactose added to provide a communal energy source [20]. After sterilization, 135 mL of culture medium was aseptically added to each of the nine fermentation vessels under anaerobic conditions. To overcome inter-individual differences in fecal microbiota and to inoculate with a diverse array of bacteria, a mixed inoculum was sourced from 10 healthy volunteers who had no known gastrointestinal disorders and had not taken any anti-, pro-, pre-, or syn-biotics for 3 months prior. The volunteers gave informed written consent to the study protocol, as approved by Northern X Regional Ethics Committee, New Zealand (13/CEN/144). The inoculum was collected in anaerobic pouches, processed within 30 min of voiding in an anaerobic chamber and stored as 20% *w/v* fecal slurries (in anaerobic glycerol buffered saline, pH 7.2) at −80 °C for a maximum of 12 months, using a previously validated protocol [21]. On the day of the experiment, the separate fecal samples were thawed in an anaerobic chamber, equi-mixed, diluted in sterile pre-reduced phosphate-buffered saline (PBS), briefly blended, and then added to each vessel at 1% *w/v*, giving a total culture volume in each vessel of 150 mL. Samples (3 mL) were taken immediately upon addition of substrate to the vessels (0 h) and then at 2.5, 5, 10, 16 and 24 h time points. Throughout the fermentation, pH was maintained at 6.8 ± 0.1 using an automated pH controller system (Fermac 260, Electrolab, Tewkesbury, UK). A range of concentrations of the hops extract were tested that related to possible concentrations that could be expected to reach the large bowel if hops-based supplements were consumed by humans. The hops samples were run at the same time as an inulin control (inulin from chicory, Sigma I-2255), a canola oil control, and an unsupplemented control, to achieve final concentrations as indicated in Table 1. All samples were run in triplicate. The hops extracts are hydrophobic and poorly digested in the human upper gut and it has been shown that many hops compounds are metabolized in the colon [10], and therefore we deemed simulated gastric-duodenal digestion unnecessary for this study.

### 2.3. Characterisation of the Microbiome

The samples were analysed by 16S rRNA gene sequencing to determine overall bacterial community composition. Total bacteria and key bacterial groups were also enumerated by qPCR to obtain absolute concentrations (method given in Supplementary Materials, with primers used for detection of key bacterial groups in Table S1, [22,23,24,25,26,27,28]). DNA was first extracted from the pellet of 1 mL fermenta using the Powersoil DNA isolation kit (MoBio Laboratories, Carlsbad, CA, USA), as per manufacturer’s instructions with the following modifications. Samples were subjected to 3 × 90-second bead beating cycles on the FastPrep-24™ 5G (MP Biomedicals, Seven Hills, Australia) at 5.5 m/s with 5 min ice rests between. A Nanodrop spectrophotometer (ND-1000, ThermoFisher) was used to determine DNA quantity and quality. DNA was submitted to the Massey Genome Service (Massey University, Palmerston North, New Zealand) where a PCR was run to amplify variable regions V3-V4 of the 16S rRNA gene using barcoded fusion primers 16SF_V3 (5′-AATGATACGGCGACCACCGAGATCTACAC-barcode-TATGGTAATTGGCCTACGGGAGGCAGCAG -3′) and 16SR_V4 (5′-CAAGCAGAAGACGGCATACGAGAT-barcode-AGTCAGTCAGCCGGACTACHVGGGTWTCTAAT-3′) [29], which also contain adaptors for downstream Illumina MiSeq sequencing. Each sample was amplified with a pair of unique (8 base) barcoded primers. The PCR conditions used were: a hold at 95 °C for 2 min followed by 30 cycles of 95 °C for 20 s, 55 °C for 15 s, 72 °C for 5 min finishing with a hold at 72 °C for 10 min. PCR reagents were Invitrogen AccuPrime™ Pfx SuperMix (Cat—12344-040) (17 µL), 10 μM 16SR_V4 Primer (1 μL), 10 μM 16SF_V3 Primer (1 μL) and 1 µL normalized sample (5 ng/µL). The PCR library clean-up kit used was an Invitrogen SequalPrep Normalisation Plate Kit (Thermo Fisher Scientific, Waltham, MA, USA). Eighteen µL of the PCR product was used in the library clean-up and the elution volume was 12 µL. A Qubit DNA High Sensitivity assay was used to measure the library concentration and a Bioanalyzer DNA High Sensitivity assay was used for library sizing. The amplicons were pooled in equal molarity and 16S rRNA gene sequencing performed on an Illumina MiSeq 2 × 250 base paired-end run.

### 2.4. Quantification of Organic Acid Metabolites

Each 1 mL sample was centrifuged at 14,000 × *g* for 10 min and the supernatant analyzed for organic acids using HPLC as previously detailed [30]. To a 0.5 mL aliquot of the supernatant, 0.6 g sodium chloride, 0.75 mL of 48% *w/v* metaphosphoric acid, and 50 mL of 1% *w/v* copper sulphate were added, and the mixture vortexed to combine. The sample was then extracted three times with diethyl ether (5 mL) containing 100 mL acetonitrile. For each extraction, samples were shaken vigorously for 10 min, followed by centrifugation at 2000× *g* for 10 min. The organic phase was transferred was then transferred to a tube containing 1 mL of 0.1 M sodium hydroxide (NaOH) and 1 drop of 2 M NaOH. The short chain fatty acids (SCFAs) were then back extracted into the alkaline aqueous phase with 10 min of shaking followed by centrifugation at 16,000× *g* at 4 °C for 20 min. The aqueous phase was transferred to a vial, following which 20 mL of 85% *w/v* orthophosphoric acid was added, followed by further mixing. The HPLC analysis was carried out using a Shimadzu LC10Avp HPLC with an SPC-10Avp UV detector (Shimadzu Co., Kyoto, Japan) and a 250 × 4.6 mm Synergi Hydro-RP 80 A column, (4 mm particle size; Phenomenex, Torrance, CA, USA). Solvents were (A) acetonitrile containing 0.025% *v/v* of 85% *w/v* orthophosphoric acid and (B) water containing 0.025% orthophosphoric acid and the flow rate was 1.0 mL/min. The initial mobile phase, 0% A, was held for 3.5 min then ramped linearly to 6% A at 20 min, 20% A at 35 min, and 50% A at 40 min before resetting to the original conditions. Sample injection volume was 20 mL. Detection was at 210 and 220 nm, and the acids were expressed as µmol organic acid/mL fermenta. Percent recovery in terms of mean ± co-efficient of variation values for formate, lactate, acetate, propionate, butyrate, isobutyrate, isovalerate and valerate were 83 ± 6, 59 ± 2, 94 ± 3, 100 ± 4, 98 ± 7, 94 ± 9, 91 ± 15, 93 ± 17, respectively. Limit of detection of these acids ranged from 0.15–0.4 µmol per mL of culture solution.

### 2.5. Bioinformatics

Quantitative Insights Into Microbial Ecology (QIIME) software version 1.8.0 was used to analyze the sequencing data [31]. To assemble the paired-end reads into a single continuous sequence, PANDASeq was used with parameters of at least 40 bp overlap, a minimum of 350 bp length and maximum of 500 bp length [32]. Putative chimeras were filtered from the sequences and the reads clustered into operational taxonomic units (OTUs) based on a 97% identity threshold value using USEARCH and UCLUST [33]. Alignment of the sequences was carried out using PyNAST [34] with reference to the Greengenes database, version 13_8 [35]. Taxonomic assignment was made using the RDP Naive Bayesian classifier [36]. Alpha diversity, using a phylogenetic-dependent method, was calculated on the rarefied data at the depth of the sample with the least reads (21,900).

### 2.6. Statistical Analysis

All statistical testing was performed in RStudio, version 3.4.2 [37] using the stats and vegan packages with the ggplot2 and qplot functions [38,39]. Adonis non-parametric tests were applied to Euclidean distance metrics to determine significance of treatments on overall genus-level community composition. Bacterial taxonomic (16S sequencing and qPCR) data and organic acid data were compared with the average control value using the non-parametric Mann–Whitney–Wilcoxon test. For all of the above analyses a *p* value < 0.05 was deemed significant after applying the false discovery rate correction for multiple comparisons. For the 16S rRNA gene sequencing only bacterial taxa present at greater than 0.5% abundance in at least one sample.

## 3. Results

The supercritical CO_2_ extract of Pacific Gem hops was found to contain α-acids and β-acids in the ratio of 1.73:1. The major α-acids were cohumulone and adhumulone while the β-acids were colupulone, lupulone and adlupulone (Table S2). 

**Figure 1:**
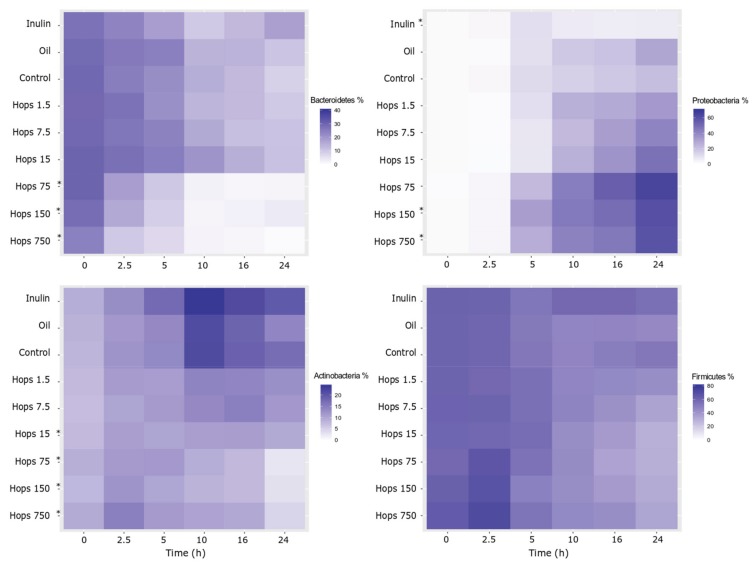
Heat plots depicting the four main phyla % relative abundance changes over time and sample type including hops concentration (mg). * *p* ≤ 0.05—Significantly different (average across all time points) compared with the control as determined by the Mann–Whitney–Wilcoxon test after false discovery rate correction.

Illumina sequencing generated over 12 million reads in 162 samples at an average of 75,194 reads per sample (minimum—21,961; maximum—238,638). The generated sequences have been deposited at https://www.ncbi.nlm.nih.gov/sra, with the Sequence Read Archive submission number # SUB6170532 [40]. Changes in microbial abundance were monitored with respect to the baseline composition, comparison with controls and changes over the subsequent sample time points. Upon interrogation of the four main phyla, it was evident that dynamic changes occurred as a result of the addition of the hops extract. Figure 1 displays the results as heat plots, where it can be seen that samples with higher hops concentrations showed a higher relative abundance of Proteobacteria and reduced abundance of Bacteroidetes, Actinobacteria and, to a lesser extent, Firmicutes. The elevated growth of Proteobacteria also occurred in the oil and unsupplemented controls but not to the degree that was evident when hops dose increased. Significant changes were found to be more prevalent at the higher hops doses whereas none were seen at the 1.5 and 7.5 mg hops concentrations (Table S3).

**Figure 2:**
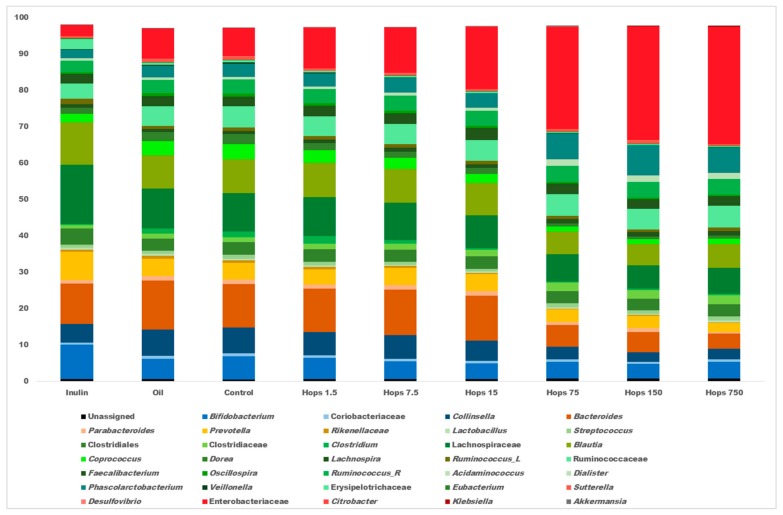
Average (*n* = 3) relative abundance of bacterial taxa in each separate fermentation (all time points). Only genera present at greater than 0.5% relative abundance in at least one sample are displayed. Genera bar colors correspond to the phyla they belong to—Actinobacteria, blue; Bacteroidetes, orange; Firmicutes, green; Proteobacteria, red; Verrucomicrobia, grey. *Ruminococcus*_R, Ruminococcaceae family; *Ruminococcus_*L, Lachnospiraceae family.

Depicted in Figure 2 are the average relative abundance values from all time points at the nearest identifiable taxonomic level, i.e., genus, family or order, where a clear dose effect was observed for many bacterial groups. Most notable was the dose-dependent increase in Enterobacteriaceae (*p* < 0.05) which consists of genera such as *Escherichia*/*Shigella*, *Enterobacter*, *Citrobacter* and *Klebsiella*. Other taxa that significantly increased at higher hops concentrations were Clostridiaceae and *Akkermansia* (Table S4). Taxa that exhibited a significant reduction in abundance as a function of elevated hops concentrations included *Bacteroides*, *Collinsella*, *Clostridium*, *Eubacterium*, *Desulfovibrio*, *Bifidobacterium*, *Blautia*, *Dorea*, *Veillonella* and Coriobacteriaceae. Alpha diversity results showed no significant alterations based on hops concentration, although a slight decrease in diversity was observed over time in all the fermentation vessels (data not shown). The canola oil control vessels had no significant differences at any taxonomic level compared with the unsupplemented control fermenters (Tables S3 and S4), which confirms the suitability of the canola oil dispersant as a non-biasing agent displaying minimal effects on the fermentation characteristics of the microbial ecosystem.

**Figure 3:**
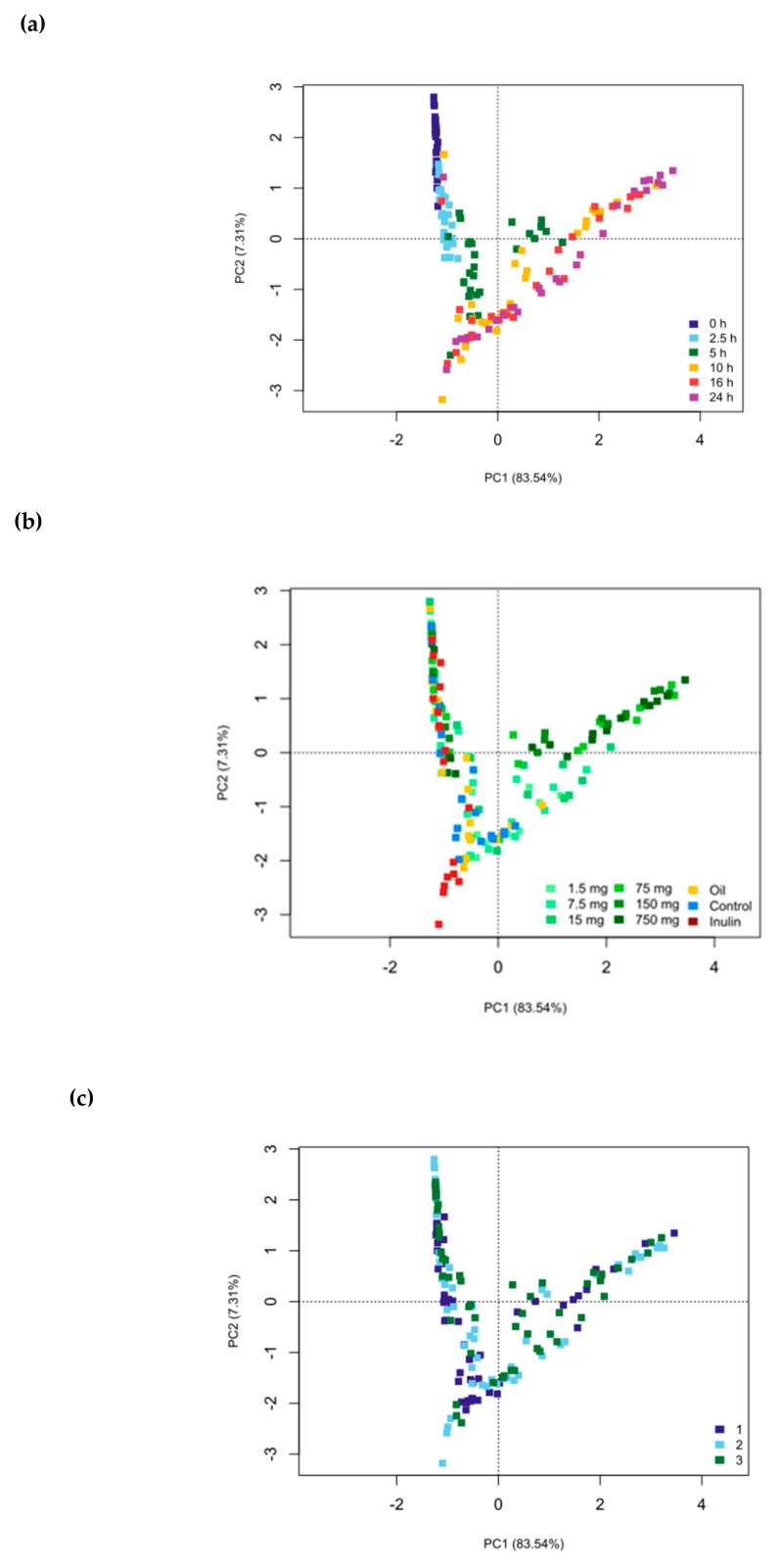
16S rRNA gene sequencing data depicted as genus-level Euclidean principal coordinates analysis (PCoA) plots separated by time point (**a**) (*p* < 0.001, R^2^ = 43.4%), substrate (**b**) (*p* < 0.001, R^2^ = 28.5%) and fermentation replicates (**c**) (*p* = 0.3248, R^2^ = 1.4%). Significance (*p* ≤ 0.05) was determined by the non-parametric Adonis statistical test (9999 permutations).

Time (*p* < 0.001, R^2^ = 43.4%) and substrate (*p* < 0.001, R^2^ = 28.5%) were most responsible for the progression of the fermentation trajectory (Figure 3). Marked changes were already evident in as little as 2.5 h, highlighting the importance of an early time point. By 5 h, bacterial populations in the higher hops concentrations (15 mg and above) had started to diverge and formed a cluster independent of the controls and inulin fermentation trajectory (Figure 3a,b. As the fermentation proceeded, the clustering became more pronounced and most disparate by 24 h. As is evident in Figure 3c, only 1.4% of the variation throughout the fermentation was accounted for over the three replicates, which was non-significant (*p* = 0.325), and confirmed minimal variation across the replicates.

To supplement the relative abundance data, selected bacterial groups were examined using qPCR giving rise to quantitative data (Table S5). The changes in total bacteria and key groups such as Lachnospiraceae, lactobacilli, bifidobacteria, *Bacteroides-Prevotella-Porphyromonas* and *Escherichia coli* revealed by qPCR were similar to those seen in the 16S rRNA gene sequencing analysis (Table S4). Compared with the control, inulin was shown to increase total bacterial numbers, the oil control did not cause a change, while the highest hops concentrations (150 and 750 mg) caused a significant decrease. Inulin also caused the expected increase in *Bifidobacterium*. Hops concentrations above 15 mg saw a significant decrease in Lachnospiraceae, and those above 75 mg caused a significant decrease in the *Bacteroides-Prevotella-Porphyromonas* group. Lactobacilli and bifidobacteria numbers were significantly diminished in all hops vessels except the lowest at 1.5 mg. *E. coli* were the only measured bacterial group to exhibit a net significant increase in the presence of the hops extract, which was observed at 150 and 750 mg.

**Figure 4:**
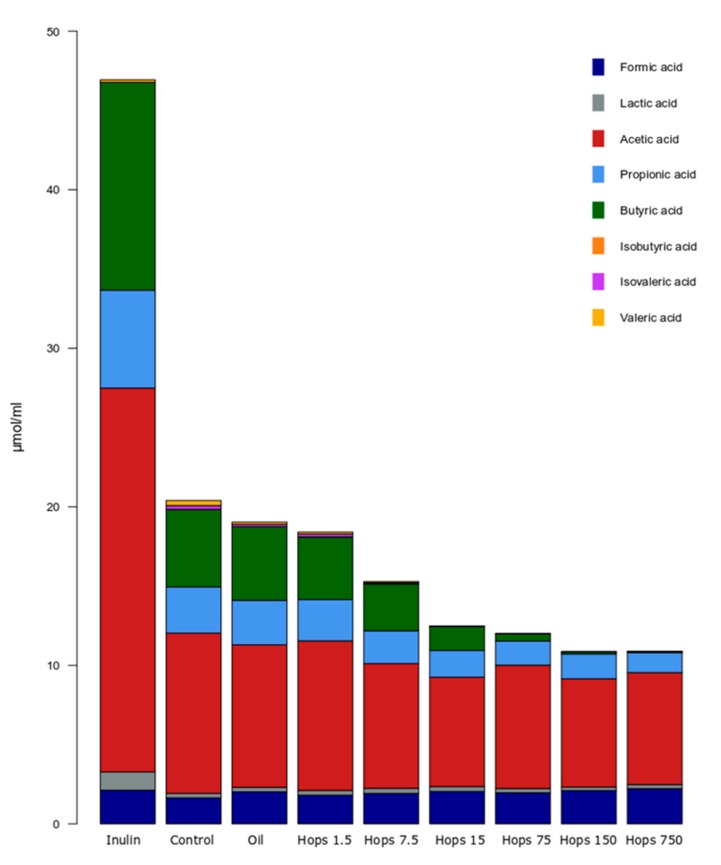
Average concentration of the measured organic acids in each separate fermentation. The substrates included hops at different concentrations (mg), oil-vehicle control, inulin and an unsupplemented control (results are the average over all time points).

The hops extract caused alterations in the concentrations of free acid metabolites in the fermentation vessels. As can be observed in Figure 4, inulin elicited a large increase in total and individual organic acid concentrations. Total organic acid amounts were comparable between the lowest hops concentration and the oil and unsupplemented controls. However, at 7.5 mg of hops and above, a consistent decrease in total organic acids was evident. Acetate and propionate concentrations did reduce slightly with higher hops concentrations, but butyrate clearly decreased when hops concentrations exceeded 7.5 mg in the fermenters. Butyrate was significantly lower than the average control values at 75, 150 and 750 mg fermentations (*p* < 0.05) (Table S6). Organic acids that also significantly decreased at higher hops levels were isovalerate and valerate, where concentrations of both acids were below the detection limit at 15 mg hops and above.

## 4. Discussion

Hops have been used as a preservative in beer brewing for centuries but also have a history of use for medicinal purposes, such as for treating anxiety, insomnia and digestive discomfort [41]. Modern day interpretations of the medical applications of hops have built upon historical uses and today they are formulated into nutraceuticals purported to aid digestion, sleeplessness and pain relief. The α-acids in hops, which cause the hop bitterness, also have demonstrated antibacterial efficacy, which has led to their use as alternatives to antibiotics in animal feed and in the brewing industry. The antimicrobial action is principally noted towards Gram-positive pathogenic bacteria, with little documented effect on Gram-negative bacteria [8,42]. The relevance of this study relates mainly to the increase in human consumption of hops-based products, but there is an increasing trend worldwide of greater hops addition in the brewing industry, both before and after boiling [43]. Indeed, prolonged consumption of these “hop forward” beers could equate to the lower hops doses investigated in this study.

As hops are likely to become a more common and more concentrated ingredient in the human diet, it is pertinent that we understand their effect on the human body and our microbial symbionts for the development of scientifically validated functional foods and nutraceuticals. The Pacific Gem hops extract contained greater α- and β-acids but were low in xanthohumol concentrations, indicating that the estrogenic aspects are not of note in this study [44]. In a pharmacological study, administration of hops at concentrations of 30–120 µM or 11 to 45 µg/mL, to intestinal cells in vitro, was calculated to correspond to an intestinal exposure that would be achieved with consumption of 10–20 mg hops in a human of 60–70 kg body weight [45,46]. Given that over-the-counter hops supplements are at a dose of 500–1000 mg, we studied concentrations up to 5000 µg/mL. Using a concentration range of the hops extract enabled us to model small concentrations of hops bitter acids through to the higher doses on the human colonic microbiota. We opted to utilize a mixed fecal inoculum in this study, which can have negative implications such as the pooling effect of multiple donors giving rise to higher diversity microbial communities and the additional drawback of the inoculum being subjected to freeze–thaw cycles [47]. However, in this case we deemed it the most appropriate approach, as individuals’ gut microbiota are highly varied, and pooling up to 10 different microbiota, can avoid the unduly high influence of any one microbial population to a substrate, as seen in other studies using individual donors [48]. The added benefit of this method was the reproducibility between experimental replicates. Another useful aspect of this study was the inclusion of a 2.5-h time point where it was seen that microbial composition was already shifting. Most in vitro fermentation studies do not incorporate such an early time point but it may be worth including to capture early microbial adaptations.

Organic acids are the most abundant end products of microbial fermentation in the colon and their production lowers luminal pH, inhibits the growth of pathogens and contributes to normal large bowel function [49]. Perhaps one of the principal markers of colonic health is butyrate, a primary energy source for colonic epithelial cells [50]. Butyrate has many beneficial effects in the large bowel, having been shown to contribute to improved intestinal barrier integrity, satiety, immune function, and is anti-inflammatory, anti-neoplastic and moderates insulin sensitivity [51,52,53]. Butyrate can be synthesized through a variety of mechanisms in the colonic environment, directly from breakdown of non-digestible carbohydrates or via metabolic cross-feeding from acetate and lactate by specific members of the microbiome [54]. In this study, we have demonstrated a dose-dependent reduction in butyrate concentration as hops concentrations increase. This may be explained by hops dose dependent decreases in key butyrate producing bacteria, *Coprococcus* and *Eubacterium*, [55,56]. or the decreases in bifidobacteria, that are metabolically versatile and able to generate butyrate through multiple pathways and metabolic cross-feeding with intestinal commensals [57]. The generation of butyrate is a functional trait not constrained to a monophyletic cluster, with most butyrate producers belonging to the Lachnospiraceae or Ruminococcaceae families [54]. Lachnospiraceae tended to drop with increasing hops concentrations, but the metabolic capacity of the Lachnospiraceae is too varied to draw meaningful conclusions from this result. Interestingly, another major butyrate producer, *Faecalibacterium*, remains relatively stable and is seemingly unaffected at elevated hops concentrations. Isovalerate and valerate were also inversely associated with hops in the gut models, becoming undetectable as hops concentrations were elevated. These organic acids are quantitatively much less abundant and therefore the consequences of their decline has less of an effect on the colonic environment. However, isovalerate production is associated with proteolytic fermentation, which can give rise to secondary metabolites such as phenols and ammonia that are detrimental to the host [58].

The bitter acids in hops have well characterized antibacterial activities, predominantly facilitated through disruption of the cellular transmembrane pH gradient [59]. The purported mechanism entails the intracellular coupling of bitter acids to divalent ions (such as Mn^2+^) and subsequent transport extracellularly, thereby dissipating the proton motive force and impairing nutrient transport and generation of energy in the form of ATP [9]. Some bacteria are resistant or tolerant to hops bitter acids (often lactic acid bacteria), typically through upregulation of transporters that actively pump hops acids out of the bacterial cell cytoplasm [60]. In our results, lactobacilli were unchanged at high hops concentrations, but it is unclear whether that is due to active resistance mechanisms. A consistent conclusion of many previous studies on hops antimicrobial effects is that Gram-positive bacteria are sensitive to hops compounds, whereas Gram-negative bacteria are unaffected. At the phylum level, Bacteroidetes and Proteobacteria, which are both composed of Gram-negative bacteria, display opposing trajectories as hops concentrations are elevated with Bacteroidetes dropping significantly and Proteobacteria increasing significantly. Actinobacteria, whose members are Gram-positive, decreased as hops concentrations rose and the predominantly Gram-positive Firmicutes were unchanged. These results suggest that Gram stain category is not necessarily indicative of hops bitter acid sensitivity/resistance, particularly using the high α-acid extract in our study. A recent study on purified hops extracts of humulone and lupulone found a potent bactericidal effect on three pure cultures of anaerobic enteric bacteria, one of which was the Gram-negative *Bacteroides fragilis* [61].

The relative abundance of *Akkermansia* increased at higher hops amounts, despite an absence of mucin polysaccharides supplemented in the media. *Akkermansia* has been shown to decrease in obese and type 2 diabetic mice [62] and researchers from the same group found hops α-acids reduced weight gain and restored glucose homeostasis in mice fed a high fat diet [63]. Further interrogation of the impact of hops compounds on individual bacterial strains is warranted to comprehend the mechanisms of hops resistance and sensitivity.

An increase in the abundance of Enterobacteriaceae was the largest magnitude modification observed in the gut models as a result of the higher hops extract. Genera within Enterobacteriaceae are not typically beneficial, as it contains members such as *Escherichia*, *Shigella*, *Citrobacter* and *Klebsiella*, some species of which are opportunistic pathogens. It should be noted that previous in vitro fermentation studies have observed elevated proportions of Enterobacteriaceae, which could be ascribed to biases in using simulated gut model systems [64,65]. High proportions of Enterobacteriaceae are sometimes observed in batch cultures, due to an eutrophication of the microbial environment with media fortified with peptone [66]. Thus, disproportional changes to the gut microbiome were seen in the control fermenter, with an increase in subdominant groups of potential pathogens and the suppression of dominant taxa of Bacteroidetes. These microbial shifts may be attributed to the much altered growth conditions in vitro (as compared to in vivo) in terms of a richer growth medium, suboptimal gaseous atmosphere (possibly more oxygen, especially during sampling, despite stringent experimental control), and the use of a static fermentation model that increases retention time and accumulation of biomolecules and wastes that are normally eliminated by absorption or excretion in vivo [64,65,66,67]. Indeed, changes in the microbiome were evidenced at 2.5 h when substrate depletion and waste accumulation are likely not a problem. 

However, an unmistakable dose-dependent augmentation of Enterobacteriaceae was observed that is distinct from the control vessels, especially with the very high concentrations of hops. This was evident both in terms of relative abundance (16S rRNA gene sequencing data) and the increase in numbers of *E*. *coli* with qPCR. However, this study was unable to determine if Enterobacteriaceae are being stimulated by hops or simply filling a niche vacated by hops-sensitive bacteria such as bifidobacteria. Irrespective, given the pathogenic potential of Enterobacteriaceae, such an effect is undesirable. Bifidobacteria are Gram-positive commensal bacteria that retard the growth of enteric pathogens by lowering intestinal pH by generation of lactate [68]. At all but the lowest hops concentration, bifidobacteria were significantly lower than that of the control in the sequencing data. Inulin is a recognized prebiotic substrate for bifidobacteria and this was evident with the significant proliferation observed in the inulin-supplemented fermenters [69]. This bifidogenic response by the positive control helped in ascertaining that the experimental conditions were adequately maintained in this in vitro model of the human colon [70]. Irrespective, we do acknowledge that an in vitro model has multiple limitations, including investigation of microbiome changes after a single dose and an inability to gauge the host gut epithelial response to microbial shifts; but this model provides a relatively rapid method to examine microbiome responses to multiple substrates, at different concentrations. In vivo models of vertebrates such as rodents, cats, dogs, monkeys while useful, have disadvantages such as a microbiome structure and composition that varies from that of humans, the pharmacological targets for the hops compounds in this project [71]. Indeed, the efficacy of nutritional intervention may be better studied using the humanized mice models i.e., gnotobiotic mice colonized with human gut bacteria [72]. Human intervention trials are thus ideal for assessing the efficacy of a nutraceutical, but challenges include the identification of a safe optimal dose range, the expense and time considerations. This in vitro model, despite its limitations provides a rapid and reliable, first-pass evaluation of the effects of the hops compounds on human gut microbiome that will help with estimation of the appropriate concentration of hops compounds and other aspects of the study design for interventional studies in humans.

## 5. Conclusions

This study established that both Gram-positive and Gram-negative gut bacteria were affected by the hop extract. This is in contrast to many other studies that show the antimicrobial activity of hops against Gram-negative bacteria is negligible. However, this may be attributed to the high α-acid content generated through supercritical CO_2_ extraction. Higher hops concentrations altered the microbial community structure by favoring the growth of Enterobacteriaceae and inhibiting beneficial probiotic bifidobacteria and butyrate-producing *Eubacterium* and *Coprococcus* genera. Furthermore, a significant dose-dependent decrease of a beneficial metabolite, butyrate, was observed as the hops extract concentration increased. In summary, our results suggest that the hops molecules exert a significant impact on microbiome structure and metabolism using in vitro fecal batch culture fermentation systems. Further research is warranted in this area, such as in vitro studies against a mixed fiber background simulating a dietary intake of fruits and vegetables and human clinical studies testing the fecal microbiome of subjects consuming a hops-supplemented diet.

## Figures and Tables

**Table 1:** Substrate concentrations in each of the nine fermentations conducted in triplicate.

Substrate	Sample Name	Hops Extract (mg) Added to Each Vessel	Final Hops Extract Concentration (µg/mL)	Inulin (mg)	Canola Oil (mg)
Hops	Hops 750	750	5000	0	375
Hops	Hops 150	150	1000	0	375
Hops	Hops 75	75	500	0	375
Hops	Hops 15	15	100	0	375
Hops	Hops 7.5	7.5	50	0	375
Hops	Hops 1.5	1.5	10	0	375
Canola Oil	Oil	0	0	0	375
Unsupplemented	Control	0	0	0	0
Inulin	Inulin	0	0	1500	0

## Supplementary Materials

The following are available online at https://www.mdpi.com/2218-273X/9/9/390/s1, Table S1: Primers and protocol for qPCR, Table S2: composition of hops extract, Table S3 and S4: microbiome abundance data, Table S5 qPCR-based enumeration of key bacterial groups, Table S6 Organic acid concentration data.
