# Supplementary material for: Dose-Dependent Alterations to In Vitro Human Microbiota Composition and Butyrate Inhibition by a Supercritical Carbon Dioxide Hops Extract
Source: Biomolecules. 2019 Aug 21;9(9):390. doi: 10.3390/biom9090390 (PMC6769549; doi:10.3390/biom9090390)
Supplement: Supplementary file 1 [file biomolecules-09-00390-s001.pdf]

biomolecules

MDPI

Appendix A

Dose-dependent alterations to *in vitro* human microbiota composition and butyrate inhibition by a supercritical carbon dioxide hops extract

Paul Blatchford[1,†], Shanthi G. Parkar[1*], Wendy Hopkins[2] , John R. Ingram[3] and Kevin H. Sutton[4]

[1] The New Zealand Institute for Plant and Food Research Limited (PFR), Private Bag 11600, Palmerston North 4442, New Zealand;

[2] PFR, Private Bag 11600, Hamilton 3240, New Zealand; wendy.hopkins@plantandfood.co.nz

[3] PFR, Auckland Mail Centre, Private Bag 92169, Auckland 1142, New Zealand; John.Ingram@plantandfood.co.nz

[4] PFR, Christchurch Mail Centre, Private Bag 4704, Christchurch 8140, New Zealand; Kevin.Sutton@plantandfood.co.nz

* Correspondence: shanthi.parkar@plantandfood.co.nz; Tel.: +64-06-953-7737(S.G.P.)

† P.A.B. current address - ZESPRI International Limited, PO Box 4043, Mt Maunganui 3149, New Zealand;Paul.Blatchford@zespri.com

Supplementary Materials and Methods

Quantitative PCR

Seven bacterial groups were quantified with selected bacteria used as representative standards for each group (Table S1). All isolates were grown anaerobically at 37°C overnight using Hungate tubes sealed with butyl rubber stoppers unless otherwise stated. All media were obtained from Oxoid (Adelaide, Australia), unless otherwise stated. *Lactobacillus reuteri* (DPC 16) was grown in de Man-Rogosa-Sharpe (MRS) broth; *Bifidobacterium bifidum* (DSM 20082) was grown in MRS broth supplemented with 0.05% (w/v) L-cysteine hydrochloride (Sigma-Aldrich); *Lachnospira multipara* (ATCC 19207) was grown in RM02 medium supplemented with filtered rumen fluid [1]; *Bacteroides fragilis* (ATCC 25285) was grown in Wilkins-Chalgren anaerobe broth supplemented with 0.05% (w/v) L-cysteine hydrochloride (Sigma-Aldrich) for 2 days; *Escherichia coli* (Nissle) was grown in tryptic soy broth (TSB) at 37°C aerobically. Cell density was determined using a Neubauer hemocytometer and cultures were diluted or concentrated as required to 1 x 10^8 or 1 x 10^9 cells/mL. DNA was then extracted using the MO-BIO PowerSoil® DNA Isolation Kit as above. Standard curves were constructed using dilution series of the bacterial strains representative of each group. Samples and standards were run in triplicate by absolute quantification on the Roche Lightcycler 480 real-time PCR instrument. Roche SYBR Green I Master Mix (04707516001) detection chemistry was used to detect double-stranded DNA amplification. Total reaction volume was 20μL, consisting of 10μL SyBr Green I Master mix, 4μL forward primer (5X concentrated at 2.5μM), 4μL reverse primer (5X concentrated at 2.5μM) and 2μL DNA template or sterile water (no template control). Each qPCR run included one activation cycle at 95°C for 5 min, 32–40 run cycles (including the denaturation step at 95°C for 30 s, annealing step as in Table A1 and extension step at 72°C for 1 min), and one melt curve cycle (60 to 95°C at 0.1°C /s with continuous fluorescence acquisition) followed by a cooling cycle at 40°C. The melt curve Tm calling cycle enabled the differentiation between target product and non-specific double-stranded product such as primer-dimers.

Table S1. Bacterial groups, standards and qPCR parameters used in this study.

Target bacteria	Bacterial standard	Primer sequence (5' → 3')	Annealing temperature (°C)	Annealing time (s)	Reference
Total bacteria	*Escherichia coli* Nissle	Fwd: TCCTACGGGAGGCAGCAGT Rev: GGACTACCAGGGTATCTAATCCTGTT	60	60	[2]
Bifidobacterium spp.	*Bifidobacterium bifidum* DSM 20082	Fwd: GGGTGGTAATGCCGGATG Rev: CCACCGTTACACCGGGAA	66	45	[3]
Lactobacillus spp.	*Lactobacillus reuteri* DPC16	Fwd: CGATGAGTGCTAGGTGTTGGA Rev: CAAGATGTCAAGACCTGGTAAG	60	30	[4]
Bacteroides-Prevotella-*Porphyromonas*	*Bacteroides fragilis* ATCC 25285	Fwd: GGTGTCGGCTTAAGTGCCAT Rev: CGGATGTAAGGGCCGTGC	63	20	[5]
Escherichia coli/Shigella spp.	*Escherichia coli* Nissle	Fwd: GAGTAAAGTTAATACCTTTGCTCATTG Rev: GAGACTCAAGCTKRCCAGTATCAG	60	20	[6]
Lachnospiraceae	*Lachnospira multipara* ATCC 19207	Fwd: GACGGTACCTGACTAAGAAGC Rev: AGTTTCATTCTTGCGAACGT	63	30	[7]

Table S2. Composition of the supercritical CO_2 hops extract used in this study as determined by High Performance Liquid Chromatography

Sample	Xanthohumol, %	Cohumulone, %	Adhumulone, %	Colupulone, %	Lupulone + Adlupulone, %	Total α-acid, %	Total β-acid, %	% α-acid as cohumulone	% β acid as colupulone	α-acid:β-acid ratio
ICE-3 standard	3	13.88	30.76	13.44	10.84	44.64	24.28	31.09	55.35	1.84
NZ Hops Pacific Gem extract	0.07	20.03	30.96	20.25	9.2	50.99	29.45	39.29	68.76	1.73

ICE-3; International Calibration Extract 3.

Table S3. Relative abundance (%) of gut microbial phyla in response to different hops concentrations (mg) and controls.

Phylum	Hops 1.5	Hops 7.5	Hops 15	Hops 75	Hops 150	Hops 750	Oil	Inulin	Unsupplemented control
Unassigned	0.7 ± 0.05	0.66 ± 0.04	0.72 ± 0.05	0.8 ± 0.06*	0.75 ± 0.05	0.81 ± 0.07	0.69 ± 0.07	0.62 ± 0.04	0.6 ± 0.03
Actinobacteria	13.28 ± 0.89	12.34 ± 0.78	10.86 ± 0.79*	9.04 ± 0.83*	7.64 ± 0.68*	8.58 ± 0.83*	13.87 ± 0.99	15.4 ± 1.14	14.58 ± 0.97
Bacteroidetes	18.37 ± 1.61	19.66 ± 1.63	19.11 ± 1.85	10.78 ± 2.25*	10.52 ± 2.2*	7.51 ± 2.16*	21.14 ± 1.71	20.93 ± 1.89	19.16 ± 1.66
Firmicutes	55.43 ± 1.56	53.68 ± 1.96	51.11 ± 2.47	49.81 ± 3.45	48.1 ± 3.33	49.6 ± 3.47	54.51 ± 1.4	59.17 ± 1.39	56.54 ± 1.25
Proteobacteria	12.04 ± 2.41	13.48 ± 2.88	18 ± 3.83	29.32 ± 5.75	32.73 ± 5.65*	33.19 ± 5.83*	9.63 ± 1.83	3.76 ± 0.57*	8.97 ± 1.53
Verrucomicrobia	0.14 ± 0.02	0.14 ± 0.02	0.16 ± 0.02	0.2 ± 0.03*	0.21 ± 0.02*	0.26 ± 0.05*	0.14 ± 0.02	0.1 ± 0.02	0.12 ± 0.02

Illumina MiSeq 16S rRNA gene sequencing data displaying phyla that were present at greater than 0.5 % relative abundance in at least one sample. Data are the calculated average values (n = 3) ± standard error of the mean (SEM) across all time points. * $p \leq 0.05$ – Significantly different compared with unsupplemented control as determined by the Mann-Whitney-Wilcoxon test after false discovery rate correction.

Table S4. Relative abundance of prevalent genera and families in response to different hops concentrations (mg) and controls.

	Hops 1.5	Hops 7.5	Hops 15	Hops 75	Hops 150	Hops 750	Oil	Inulin	Control
Unassigned	0.7 ± 0.05	0.66 ± 0.04	0.72 ± 0.05	0.8 ± 0.06*	0.75 ± 0.05	0.81 ± 0.07	0.69 ± 0.07	0.62 ± 0.04	0.6 ± 0.03
Bifidobacterium	5.77 ± 0.32	4.87 ± 0.29*	4.29 ± 0.33*	4.66 ± 0.46*	4.08 ± 0.36*	4.62 ± 0.46*	5.59 ± 0.34	9.56 ± 0.96*	6.29 ± 0.4
Coriobacteriaceae	0.77 ± 0.04	0.68 ± 0.03*	0.62 ± 0.04*	0.58 ± 0.06*	0.53 ± 0.05*	0.6 ± 0.05*	0.75 ± 0.04	0.55 ± 0.03*	0.82 ± 0.05
Collinsella	6.41 ± 0.74	6.49 ± 0.72	5.67 ± 0.67	3.48 ± 0.36*	2.73 ± 0.25*	3.02 ± 0.29*	7.21 ± 0.75	5.09 ± 0.32	7.15 ± 0.77
Bacteroides	11.88 ± 0.45	12.57 ± 0.54	12.29 ± 0.78	5.97 ± 0.95*	5.57 ± 0.91*	4.1 ± 0.95*	13.53 ± 0.84	11.1 ± 0.83	11.91 ± 0.62
Parabacteroides	1.08 ± 0.07	1.28 ± 0.13	1.19 ± 0.1	0.85 ± 0.15*	1.11 ± 0.2	0.43 ± 0.08*	1.28 ± 0.1	0.94 ± 0.06*	1.26 ± 0.09
Prevotella	4.25 ± 1.39	4.68 ± 1.37	4.72 ± 1.47	3.45 ± 1.25	3.31 ± 1.27	2.55 ± 1.19	4.69 ± 1.34	7.84 ± 1.82	4.62 ± 1.27
Rikenellaceae	0.6 ± ± 0.11	0.5 ± 0.09	0.39 ± 0.03*	0.2 ± 0.04*	0.19 ± 0.04*	0.1 ± 0.03*	0.88 ± 0.17	0.56 ± 0.08	0.73 ± 0.12
Lactobacillus	0.45 ± 0.04	0.39 ± 0.04	0.35 ± 0.04	0.47 ± 0.07	0.38 ± 0.05	0.51 ± 0.07	0.44 ± 0.04	0.49 ± 0.05	0.4 ± 0.04
Streptococcus	0.96 ± 0.07	0.78 ± 0.06	0.78 ± 0.07	1.03 ± 0.12	0.91 ± 0.09	1.12 ± 0.11	0.95 ± 0.06	0.89 ± 0.11	1.01 ± 0.08
Clostridiales	3.45 ± 0.3	3.38 ± 0.3	3.43 ± 0.34	3.34 ± 0.39	3.22 ± 0.44	3.31 ± 0.42	3.32 ± 0.25	4.41 ± 0.13*	3.53 ± 0.23
Clostridiaceae	1.59 ± 0.17	1.67 ± 0.18	1.78 ± 0.17	2.38 ± 0.18*	2.38 ± 0.17*	2.59 ± 0.22*	1.38 ± 0.16	0.97 ± 0.2*	1.42 ± 0.16
Clostridium	2.11 ± 0.61	0.95 ± 0.22	0.38 ± 0.05*	0.34 ± 0.04*	0.32 ± 0.04*	0.36 ± 0.05*	1.38 ± 0.36	0.23 ± 0.03*	1.49 ± 0.34
Lachnospiraceae	10.63 ± 0.68	10.2 ± 0.73	9.08 ± 0.73	7.41 ± 1.01	6.43 ± 1.11*	7.06 ± 1.07	10.98 ± 0.67	16.32 ± 1.34*	10.56 ± 0.71

Blautia	9.42 ± 0.52	9.36 ± 0.55	8.86 ± 0.56	6.29 ± 0.81*	5.85 ± 0.74*	6.58 ± 0.65*	9.18 ± 0.55	11.6 ± 0.88	9.37 ± 0.53
Coprococcus	3.5 ± 0.38	2.99 ± 0.33	2.54 ± 0.24*	1.43 ± 0.27*	1.36 ± 0.29*	1.48 ± 0.27*	3.78 ± 0.37	2.48 ± 0.15*	4.06 ± 0.4
Dorea	1.95 ± 0.23	1.77 ± 0.24	1.58 ± 0.2	0.83 ± 0.08*	0.76 ± 0.08*	0.91 ± 0.08*	2.5 ± 0.35	1.58 ± 0.13	2.83 ± 0.4
Lachnospira	0.97 ± 0.17	1.01 ± 0.16	1.06 ± 0.17	1.21 ± 0.18	1.17 ± 0.17	1.18 ± 0.15	0.88 ± 0.15	0.94 ± 0.16	0.89 ± 0.15
Ruminococcus_L	0.97 ± 0.07	0.98 ± 0.06	0.97 ± 0.07	0.85 ± 0.09	0.81 ± 0.08	0.95 ± 0.08	0.91 ± 0.06	1.62 ± 0.2*	0.95 ± 0.07
Ruminococcaceae	5.47 ± 0.56	5.67 ± 0.53	5.7 ± 0.53	5.98 ± 0.66	5.61 ± 0.66	6.08 ± 0.66	5.38 ± 0.49	4.18 ± 0.66	5.81 ± 0.42
Faecalibacterium	2.87 ± 0.58	2.88 ± 0.55	3.31 ± 0.6	2.91 ± 0.6	2.72 ± 0.62	2.64 ± 0.61	2.82 ± 0.58	2.55 ± 0.55	2.62 ± 0.54
Oscillospira	0.66 ± 0.08	0.64 ± 0.07	0.55 ± 0.08*	0.45 ± 0.09*	0.42 ± 0.09*	0.43 ± 0.09*	0.73 ± 0.07	0.46 ± 0.07*	0.87 ± 0.09
Ruminococcus_R	3.88 ± 0.4	4.25 ± 0.38	4.18 ± 0.38	4.42 ± 0.52	4.23 ± 0.53	4.21 ± 0.43	3.6 ± 0.39	3.25 ± 0.48	3.84 ± 0.43
Acidaminococcus	0.11 ± 0.02	0.11 ± 0.03	0.1 ± 0.03	1.1 ± 0.54	1.22 ± 0.61	1.07 ± 0.55	0.09 ± 0.02	0.1 ± 0.02	0.1 ± 0.02
Dialister	0.69 ± 0.17	0.74 ± 0.19	0.69 ± 0.16	0.65 ± 0.18	0.53 ± 0.16	0.69 ± 0.17	0.64 ± 0.16	0.54 ± 0.16	0.66 ± 0.15
Phascolarctobacterium	3.62 ± 0.55	4 ± 0.67	4.05 ± 0.85	7.17 ± 1.36	8.37 ± 1.68	6.95 ± 1.39	3.25 ± 0.52	2.43 ± 0.32	3.59 ± 0.47
Veillonella	0.23 ± 0.04	0.12 ± 0.01*	0.08 ± 0.01*	0.13 ± 0.03*	0.09 ± 0.01*	0.08 ± 0.01*	0.27 ± 0.1	0.12 ± 0.02*	0.4 ± 0.08
Erysipelotrichaceae	0.41 ± 0.04*	0.43 ± 0.05	0.4 ± 0.05*	0.25 ± 0.05*	0.24 ± 0.04*	0.32 ± 0.05*	0.53 ± 0.08	2.73 ± 0.44*	0.61 ± 0.07
Eubacterium	0.07 ± 0.01*	0.07 ± 0.01*	0.07 ± 0.01*	0.05 ± 0.01*	0.05 ± 0.01*	0.05 ± 0.01*	0.1 ± 0.02	0.22 ± 0.04	0.12 ± 0.02
Sutterella	0.65 ± 0.12	0.63 ± 0.14	0.52 ± 0.08	0.7 ± 0.14	0.95 ± 0.2	0.4 ± 0.09*	0.93 ± 0.14	0.61 ± 0.08	0.84 ± 0.14

Desulfovibrio	0.03 ± 0*	0.03 ± 0.01*	0.02 ± 0*	0.1 ± 0.04	0.08 ± 0.03	0.02 ± 0*	0.04 ± 0.01	0.02 ± 0*	0.06 ± 0.01
Enterobacteriaceae	11.08 ± 2.43	12.5 ± 2.9	17.1 ± 3.81	28.02 ± 5.69	31.2 ± 5.56*	32.27 ± 5.8*	8.37 ± 1.81	2.99 ± 0.56	7.8 ± 1.55
Citrobacter	0.05 ± 0.02	0.07 ± 0.03	0.11 ± 0.05	0.08 ± 0.03	0.05 ± 0.02	0.04 ± 0.02	0.04 ± 0.02	0 ± 0	0.03 ± 0.02
Klebsiella	0 ± 0	0.01 ± 0	0.01 ± 0.01	0.07 ± 0.04	0.12 ± 0.06	0.17 ± 0.08	0 ± 0	0 ± 0	0 ± 0
Akkermansia	0.14 ± 0.02	0.14 ± 0.02	0.16 ± 0.02	0.2 ± 0.03*	0.21 ± 0.02*	0.26 ± 0.05*	0.14 ± 0.02	0.1 ± 0.02	0.12 ± 0.02

Illumina MiSeq sequencing data displaying taxa that were present at greater than 0.5% relative abundance in at least one sample. Data are the calculated average values (n = 3) ± standard error of the mean (SEM) across all time points. * $p \leq 0.05$ – Significantly different compared with control as determined by the Mann-Whitney-Wilcoxon test after false discovery rate correction.

Table S5 Average 16S rRNA gene copies/mL (log transformed) of select bacterial groups in response to different hops concentrations (mg) and controls across all time points.

Bacterial group	Hops 1.5	Hops 7.5	Hops 15	Hops 75	Hops 150	Hops 750	Oil	Inulin	Control
Lachnospiraceae	7.96 ± 0.05	7.91 ± 0.05	7.77 ± 0.04*	7.48 ± 0.07*	7.34 ± 0.10*	7.33 ± 0.09*	8.01 ± 0.05	8.25 ± 0.08	8.05 ± 0.05
Bacteroides-Prevotella-Porphyromonas.	7.81 ± 0.03	7.81 ± 0.03	7.73 ± 0.04	7.24 ± 0.08*	7.23 ± 0.10*	6.81 ± 0.14*	7.9 ± 0.03	7.92 ± 0.08	7.83 ± 0.03
Bifidobacteria	7.44 ± 0.07	7.33 ± 0.05*	7.19 ± 0.04*	7.1 ± 0.03*	6.99 ± 0.06*	6.96 ± 0.05*	7.47 ± 0.06	7.82 ± 0.11	7.55 ± 0.07
Lactobacillus	7.29 ± 0.02	7.19 ± 0.02*	7.12 ± 0.04*	7.08 ± 0.04*	7.01 ± 0.07*	6.99 ± 0.07*	7.31 ± 0.02	7.29 ± 0.04	7.33 ± 0.02
Escherichia coli	8.14 ± 0.22	8.14 ± 0.23	8.21 ± 0.24	8.56 ± 0.29	8.58 ± 0.28*	8.56 ± 0.28*	8.09 ± 0.20	7.89 ± 0.17	8.11 ± 0.20
Total bacteria	9.56 ± 0.05	9.52 ± 0.05	9.46 ± 0.05	9.49 ± 0.06	9.42 ± 0.06*	9.33 ± 0.05*	9.58 ± 0.05	9.74 ± 0.07	9.61 ± 0.05

Quantitative PCR data displaying select bacterial groups. Data are the calculated average values ($n = 3$) ± standard error of the mean (SEM). * $P \leq 0.05$ – Significantly different compared with the control as determined by the Mann-Whitney-Wilcoxon test after false discovery rate correction.

Table S6. Average concentrations (μmol/mL) of principal organic acids in response to different hops concentrations (mg) and controls.

	**Hops 1.5**	**Hops 7.5**	**Hops 15**	**Hops 75**	**Hops 150**	**Hops 750**	**Oil**	**Inulin**	**Control**
Formic acid	1.81 ± 0.22	1.92 ± 0.22	2.04 ± 0.25	1.96 ± 0.28	2.1 ± 0.24	2.22 ± 0.3	2.02 ± 0.27	2.12 ± 0.32	1.64 ± 0.22
Lactic acid	0.3 ± 0.07	0.28 ± 0.05	0.31 ± 0.05	0.27 ± 0.02	0.22 ± 0.01	0.25 ± 0.01	0.28 ± 0.08	1.15 ± 0.33	0.28 ± 0.08
Acetic acid	9.43 ± 1.83	7.87 ± 1.46	6.9 ± 1.41	7.78 ± 1.77	6.83 ± 1.53	7.07 ± 1.62	8.99 ± 1.58	24.22 ± 4.27	10.11 ± 1.8
Propionic acid	2.6 ± 0.56	2.07 ± 0.49	1.69 ± 0.39	1.52 ± 0.35	1.56 ± 0.37	1.26 ± 0.3	2.81 ± 0.57	6.18 ± 1.48	2.91 ± 0.58
Butyric acid	3.96 ± 1.3	2.96 ± 1.09	1.53 ± 0.51	0.46 ± 0.16*	0.14 ± 0.06*	0.07 ± 0.05*	4.64 ± 1.51	13.11 ± 3.14	4.9 ± 1.43
Isobutyric acid	0 ± 0	0 ± 0	0 ± 0	0 ± 0	0 ± 0	0 ± 0	0 ± 0	0 ± 0	0 ± 0
Isovaleric acid	0.18 ± 0.08	0.07 ± 0.05	0 ± 0*	0 ± 0*	0 ± 0*	0 ± 0*	0.15 ± 0.09	0 ± 0*	0.24 ± 0.1
Valeric acid	0.13 ± 0.08	0.08 ± 0.06	0 ± 0*	0 ± 0*	0 ± 0*	0 ± 0*	0.15 ± 0.09	0.15 ± 0.06	0.32 ± 0.12
Total	18.41 ± 3.4	15.29 ± 2.82	12.47 ± 2.29	11.99 ± 2.31	10.86 ± 2	10.87 ± 1.97	19.04 ± 3.29	46.92 ± 8.61	20.4 ± 3.5

HPLC data of organic acids displayed as the calculated average values (n = 3) ± standard error of the mean (SEM) across all time points. * $P \leq 0.05$ – Significantly different compared with control as determined by the Mann-Whitney-Wilcoxon test after false discovery rate correction.

References

1. Leahy, S.C.; Kelly, W.J.; Altermann, E.; Ronimus, R.S.; Yeoman, C.J.; Pacheco, D.M.; Li, D.; Kong, Z.H.; McTavish, S.; Sang, C., *et al.* The genome sequence of the rumen methanogen *Methanobrevibacter ruminantium* reveals new possibilities for controlling ruminant methane emissions. *PLoS ONE* **2010**, *5*, e8926.
2. Nadkarni, M.A.; Martin, F.E.; Jacques, N.A.; Hunter, N. Determination of bacterial load by real-time PCR using a broad-range (universal) probe and primers set. *Microbiology* **2002**, *148*, 257-266.
3. Kok, R.G.; DeWaal, A.; Schut, F.; Welling, G.W.; Weenk, G.; Hellingwerf, K.J. Specific detection and analysis of a probiotic *Bifidobacterium* strain in infant feces. *Appl. Environ. Microbiol.* **1996**, *62*, 3668-3672.
4. Fu, C.J.; Carter, J.N.; Li, Y.; Porter, J.H.; Kerley, M.S. Comparison of agar plate and real-time PCR on enumeration of *Lactobacillus*, *Clostridium perfringens* and total anaerobic bacteria in dog faeces. *Lett. Appl. Microbiol.* **2006**, *42*, 490-494.
5. Rinttila, T.; Kassinen, A.; Malinen, E.; Krogius, L.; Palva, A. Development of an extensive set of 16S rDNA-targeted primers for quantification of pathogenic and indigenous bacteria in faecal samples by real-time PCR. *J. Appl. Microbiol.* **2004**, *97*, 1166-1177.
6. Kurakawa, T.; Kubota, H.; Tsuji, H.; Matsuda, K.; Takahashi, T.; Ramamurthy, T.; Nair, G.B.; Takeda, Y.; Nomoto, K. Intestinal *Enterobacteriaceae* and *Escherichia coli* populations in Japanese adults demonstrated by the reverse transcription-quantitative PCR and the clone library analyses. *J. Microbiol. Methods* **2013**, *92*, 213-219.
7. Paturi, G.; Butts, C.A.; Bentley-Hewitt, K.L.; Ansell, J. Influence of green and gold kiwifruit on indices of large bowel function in healthy rats. *J. Food Sci.* **2014**, *79*, H1611-H1620.
